# Fisheries-induced life-history changes recover in experimentally harvested fish populations

**DOI:** 10.1098/rsbl.2024.0319

**Published:** 2024-11-06

**Authors:** Stephan N. van Dijk, Daniel E. Sadler, Phillip C. Watts, Silva Uusi-Heikkilä

**Affiliations:** ^1^ Department of Biological and Environmental Science, University of Jyväskylä, Jyväskylä, Finland

**Keywords:** fisheries, experimental, size-selective harvesting, life-history traits, generational

## Abstract

Overfishing is one of the greatest threats to fish populations. Size-selective harvesting favours faster juvenile growth, younger maturation, small adult body size and low reproductive output. Such changes might be slow to recover and ultimately threaten population fitness and survival. To study the recovery potential of exploited experimental populations, we compared life-history traits in three differently size-selected experimental lines (large-selected, small-selected and random-selected) after five generations of harvesting and 10 subsequent generations of recovery (i.e. cessation of harvesting). We show that after a recovery period twice as long as the harvesting period, the differences in adult body size among the selection lines have eroded. While there was still a significant body size difference among the selection lines, this did not translate to differences in reproductive success. Although size-selective harvesting causes phenotypic changes in exploited fish populations, we show that such changes are reversible if the recovery period is long enough.

## 1. Introduction

Many fisheries exert size-selective harvesting [1,2], and such selective removal of the larger individuals can cause marked phenotypic [3,4] and genetic [5,6] changes in the population. Size-selective harvesting promotes traits, such as faster juvenile growth, early maturation, smaller adult body size, lower schooling cohesion and fecundity compared with the effects of a random (with regard to phenotype) reduction in population size [1,4,7–10]. Fisheries-induced phenotypic changes can develop rapidly, for example, within three generations in experimental models of size-selective harvesting depending on the strength of the selection [8]. However, it has been suggested that it takes many generations for a certain average phenotypic trait value to revert toward that of the ancestral phenotype following the cessation of harvesting (a ‘phenotypic recovery’) [11–16], though empirical evidence of full phenotypic recovery is scarce.

Quantifying the rate of phenotypic recovery after harvest-induced phenotypic change is important for management and conservation efforts. For example, this information can reveal the mechanisms underlying fisheries-induced change, as phenotypic changes with a predominantly plastic component may be expected to recover relatively rapidly [17,18], whereas it is predicted that it can take 10 or more generations for a phenotypic recovery when the phenotype has a genetic basis [1,19,20].

As size-selective harvesting can cause genetic changes over contemporary timescales [5,6,9,21–23], one might expect harvested populations to have a slow phenotypic recovery, especially when the phenotypic changes are accompanied by genetic changes at growth-associated loci. A slow rate of phenotypic recovery may be expected as many of the phenotypic changes induced by size-selective harvesting (such as small adult body size and low fecundity) can oppose natural selection to negatively affect key life-history traits such as reproductive output and survivability [7,12]. However, if selective pressures, such as intrinsic fecundity selection [24], are strong enough to oppose the (previous) size selection, the exploited population could revert toward its original phenotype.

No previous research has demonstrated a full phenotypic recovery. In prior research by Conover *et al*. [12], four generations of size-selective harvesting (with 90% mortality rate) were sufficient to induce phenotypic changes in experimental populations of the Atlantic silverside (*Menidia menidia*)—lines of small size-selected fish were, on average, 50% (12 mm) smaller than fish from the control lines. After five generations without harvesting, the size-selected lines exhibited a 50% phenotypic recovery (i.e. only 6 mm smaller than fish from control lines) [12]. Conover *et al*. hypothesized that fish stocks may have an intrinsic capacity to recover from the phenotypic changes caused by size-selective fishing. However, they also noted that since at least some commercially exploited fish species (e.g. cod, halibut and tuna) have long generation times, the phenotypic recovery can take decades, but this has not been studied in the wild. Extrapolated from their experimental data, Conover *et al*. [12] predicted that the time for full phenotypic recovery (measured in generations) would be three times longer than the harvesting period. However, their prediction was based on extrapolating their experimental data after five generations without size-selective harvesting. As no empirical data of the recovery rates of fully recovered size-selectively harvested populations exist, we studied this experimentally using a model species. However, we predict that a recovery period twice the number of generations under size-selective harvesting would be sufficient to show a significant, if not complete, return to the previous phenotypic baseline given, for example, potentially strong effect of intrinsic fecundity selection in zebrafish [25].

To quantify the recovery potential of exploited experimental populations, we compared life-history traits in three zebrafish (*Danio rerio*) lines that had been harvested for five generations and allowed to recover (i.e. no harvesting) for 10 subsequent generations. Previous research using the same model system has focused on changes in behaviour [10,26,27], reproduction and mate choice [9,25,28], trait variability [9,29], cognition [30], circadian system [31] and the genomic structure [9,22,23,32] after the cessation of harvesting. Here, we focus on a range of life-history traits 10 generations after the cessation of size-selective harvesting. During the harvesting period, two lines experienced size-selective harvesting for either small or large body size, and the control line was harvested randomly with respect to body size. Five generations of harvesting induced significant phenotypic differences among the selection lines: fish selected for small body size were smaller (7.6% and 7.2% smaller juveniles and adults, respectively), matured 15 days earlier and at a smaller size (7.6% smaller), invested relatively more energy in reproduction (23.5% more) but produced fewer eggs (38.6% less) than large-selected fish [9]. Random-selected fish were phenotypically not significantly different from large-selected fish, except for age at maturity (15 days earlier) and relative fecundity (39.3% less eggs), in which they were more akin to small-selected fish [9]. Furthermore, the experimental harvesting induced genomic differences among the selection lines [9,22,23]. Given the harvest-induced genomic differences among the selection lines are at least partly associated with the phenotypic differences, one could expect that the phenotypic differences among the lines would not be fully eroded after 10 generations of recovery. On the other hand, fish size-related life-history traits are also known to be plastic [17,18], and the intrinsic fecundity selection can be strong after the cessation of harvesting [25]. Therefore, phenotypic differences among the lines could be expected to be eroded to some degree after 10 generations of recovery.

## 2. Material and methods

### (a) Harvesting design

The zebrafish founder population originated from the West Bengal region of India, and the fish were transported to the Institute of Freshwater Ecology and Inland Fisheries, Berlin, Germany [25]. The fish were subjected to three harvesting treatments with two replicates each: small-selected (75% of the largest fish harvested; mimicking size-selective fisheries), large-selected (75% of the smallest fish harvested) and random-selected (75% of the population harvested randomly regarding body size; the control line). Harvesting continued for five generations (electronic supplementary material, figure S1), after which significant phenotypic and genomic differences among the selection lines were demonstrated [9,22,23,32]. After five generations of harvesting, the fish were maintained for another three generations with no harvesting in Berlin and then transported to Finland, where the recovery period continued for another seven generations. This no-harvesting period is referred to as a recovery period (electronic supplementary material, figure S1).

### (b) Phenotypic measurements

After 10 generations of recovery, we monitored various phenotypic traits of the zebrafish selection lines. Adult body size was measured at age 210 days post-fertilization (dpf). The individual growth rate was calculated as


$$(({SL}_{2}-{SL}_{1})/t)\times 100$$

where SL_2_ is the average standard length (SL (mm); or wet mass, WM (g)) at the end of the experiment, SL_1_ is the average SL (or WM) at the start of the experiment and *t* is the time the growth experiment lasted in days.

Differences in reproductive success among the selection lines were assessed in spawning trials that lasted for 5 days. We monitored spawning probability, number of eggs produced per female per day (clutch size), egg fertilization rate, egg size (mm), egg mortality rate, larval size-at-hatch (SL, mm) and larval yolk sac size (mm). More details about the methodology can be found in the electronic supplementary material.

### (c) Statistical analyses

All statistics were performed in R v. 4.1.2 [33] using the *lmer* and *glmer* functions from the lme4 package [34] and *lmertest* from the lmerTest package [35]. We used linear mixed models to analyse differences in adult body size (SL and WM), growth rate, fertilization rate, egg yolk size, larval age-at-hatch, larval size-at-hatch and yolk sac size among the selection lines. We used a generalized linear mixed model with binomial error distribution for the spawning probability and with Poisson error distribution for the clutch size. We ensured that assumptions of homogeneity and normality of residuals were met for each response variable. In all statistical analyses, the selection line was used as a fixed effect and selection-line replicate, spawning day, tank (in spawning experiment) and rearing container (in growth experiment) as random effects (table 1). ANOVA was used to determine the most parsimonious model using stepwise model reduction (table 1). Tukey’s range test was used to determine if any statistically significant differences were found between the selection lines.

**Table 1.** Least complicated models and TukeyHSD posthoc test results to compare the differences between the selection lines. *:*p*-Value = 0.05–0.01, **:*p*-value = 0.01–0.001, ***:*p*-value < 0.00

trait	starting model	least complicated model	comparison	estimate	*z*/*t* value	*p*‐value
standard length (SL; cm)	SL ~ Selection_line + (1|tank) + (1|replicate) + (1|cage)	SL ~ Selection_line	small–large	−0.09777	−0.198	0.97852
			small–random	1.49393	3.007	0.00946 **
			large–random	1.5917	3.156	0.00612 **
wet mass (WM; g)	WM ~ Selection_line + (1|Tank) + (1|Replicate) + (1|Cage)	WM ~ Selection_line	small–large	0.01155	0.965	0.601
			small – random	0.02282	1.891	0.147
			large–random	0.01127	0.92	0.629
clutch size	Clutch_size ~ Selection_line + (1| Replicate) + (1|Box) + (1|Date)	Clutch_Size ~ Selection_line + (1| Replicate) + (1|Box) + (1|Date)	small–large	−0.6459	−2.932	0.0947 **
			small–random	−0.1669	−0.762	0.72654
			large–random	0.479	0.218	0.07464
fertilization rate	Fertilization_rate ~ Selection_line + (1| Replicate) + (1|Box) + (1|Date)	Fertilization_rate ~ Selection_line + (1|Box) + (1|Date)	small–large	−0.7702	−4.996	<1e−04 ***
			small–random	−0.2838	−1.863	<1e−04 ***
			large–random	0.4864	3.139	0.00475 **
spawning probability	Spawning ~ Selection_line + (1| Replicate) + (1|Week)	Spawning ~ Selection_line + (1| Replicate)	small–large	25	1.78	0.1762
			small–random	32.143	2.289	0.0573
			large–random	7.143	0.509	0.8671
larval size at hatch	Larva_length ~ Selection_line + (1| Replicate) + (1|Well) + (1|Hatching_Date)	Larva_length ~ Selection_line + (1| Replicate) + (1|Hatching_Date)	small–large	0.0784	0.873	0.657
			small–random	0.04629	0.546	0.848
			large–random	−0.03211	−0.382	0.923
yolk sac diameter	Log(Yolk sac) ~ Selection_line + (1| Replicate) + (1|Well) + (1|Hatching_Date)	Log(Yolk sac) ~ Selection_line + (1| Replicate) + (1|Hatching_Date)	small–large	0.043125	0.764	0.735
			small–random	0.04974	0.918	0.629
			large–random	0.06616	0.124	0.992
egg yolk diameter	Egg yolk ~ Selection_line + (1| Replicate) + (1|Well) + (1|Hatching_Date)	Egg yolk ~ Selection_line + (1| Replicate) + (1|Hatching_Date)	small–large	0.01153	0.811	0.6963
			small–random	−0.01849	−1.316	0.3859
			Large–random	−0.03002	−2.186	0.0735
growth rate wet mass	WM_growth ~ Selection_line + (1|Tank) + (1|Replicate) + (1|Cage)	WM_growth ~ Selection_line	small–large	0.03515	0.673	0.779
			small–random	0.09431	1.792	0.178
			large–random	−0.05916	−1.107	0.512
growth rate standard length	SL_growth ~ Selection_line + (1|Tank) + (1|Replicate) + (1|Cage)	SL_growth ~ Selection_line	small–large	−1.651	−0.6	0.8204
			small–random	6.624	2.387	0.0494 *
			large–random	8.275	2.938	0.115 *

## 3. Results and discussion

**Figure 1.**
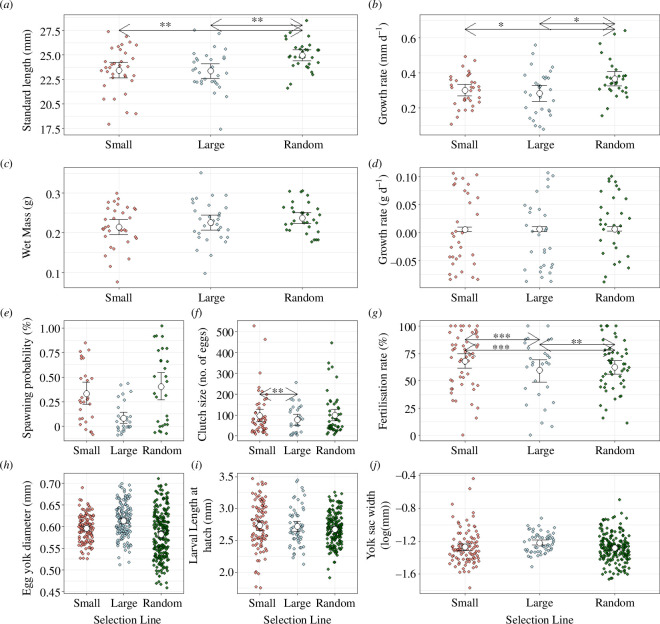
Variation in life-history traits among the small-selected (red), large-selected (blue) and random-selected (green) lines after 10 generations of recovery (selection-line replicates combined). (*a*) Standard length of the individuals at age 210 dpf (*N* = 120), (*b*) growth rate in length per day (*N* = 120), (*c*) wet mass of the individuals at age 210 dpf (*N* = 120), (*d*) growth rate in mass per day (*N* = 120), (*e*) spawning probability (*N* = 84), (*f*) number of fertilized eggs produced per female per spawning event (*N* = 130), (*g*) egg fertilization rate (*N* = 130), (*h*) egg size (*N* = 517), (*i*) larval length-at-hatch (*N* = 347) and (*j*) larval yolk sac width (*N* = 344). Data are shown as individual observations per fish (dots) and the mean with standard error across selection line replicates. *p-Value = 0.05–0.01, **p-value = 0.01–0.001, ***p-value < 0.001.

**Figure 2.**
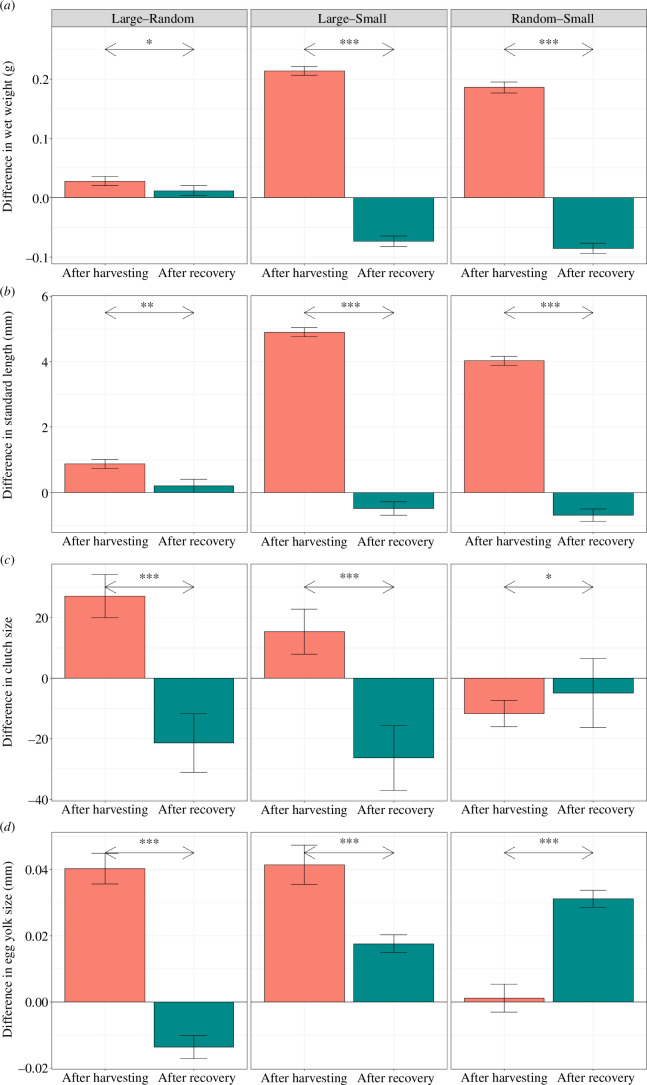
Relative differences in life-history traits between the differently size-selected fish after harvesting (red) and after recovery (green). (*a*) Wet mass, (*b*) standard length of the individuals at age 210 dpf, (*c*) number of fertilized eggs per female per one spawning event and (*d*) egg yolk size. A positive value indicates that the average trait value was greater among the selection line mentioned on the left of the header than the selection line on the right. A negative value indicates that the average trait value was smaller among the selection line mentioned on the left of the header than the selection line on the right. Bars indicate means with standard error across line replicates. *p-Value = 0.05–0.01, **p-value = 0.01–0.001, ***p-value < 0.001.

After 10 generations of recovery, there were no significant differences in adult body size (*p* > 0.05) or growth rate (*p* > 0.05; figure 1*a–d*; table 1) between small- and large-selected fish, although 10 generations earlier (i.e. after five generations of size-selective harvesting) small-selected fish were smaller (on average 4.9 mm) than large- and random-selected fish (*p* < 0.001; figure 2*a*,*b*; electronic supplementary material, figure S2*a*,*b*). After 10 generations of recovery, random-selected fish were significantly larger (1.5 mm or 6.4%; *p* < 0.05; figure 1*a*) and had a significantly faster growth rate compared to the size-selected lines (*p* < 0.05; figure 1*b*; table 1).

Small-selected fish likely increased in body size during the recovery period, rather than large- and random-selected fish decreased. In nature, small-sized fish may have lower fitness than large-sized fish as they can be less capable of escaping predators and are affected more by limited resources [36,37]. However, in laboratory conditions, there are no predators, and fish are provided with plenty of resources. Because large-sized fish generally have higher reproductive fitness than small-sized fish [25,38,39], intrinsic fecundity selection may, therefore, explain the gradual loss of phenotypic differences among the selection lines and also support the hypothesis that instead large- and random-selected fish becoming smaller after the size-selective harvesting had ceased, small-selected fish were actually becoming larger.

There were no significant differences in most of the measured reproductive traits among the selection lines (figure 1*e*–*j*; table 1). Before the recovery period, small-selected fish had significantly lower spawning probability and smaller clutch size than large- and random-selected fish (*p* < 0.05; figure 2*c*). Large-selected fish also produced significantly larger eggs than small- and random-selected fish (*p* < 0.01; Figure 2*d*). However, after the recovery period, small-selected fish had a significantly higher clutch size than large-selected fish, but not random-selected (*p* < 0.05; figure 1*f*; table 1). In addition, the egg fertilization rate was significantly higher among the small-selected fish compared to large- and random-selected fish (*p* < 0.005; figure 1*g*; table 1). Despite no difference in body size, small-selected fish had higher reproductive output than large- and random-selected fish as they produced more eggs with higher fertilization rate. This is possibly a holdover from the previous selective pressure. After five generations of harvesting, small-selected fish had higher reproductive investment but lower reproductive success (spawning probability and clutch size) than the other selection lines [9]. This means that small-selected fish were allocating more energy into reproduction earlier in life than large- and random-selected fish, which were instead allocating energy into somatic growth. Life-history theory predicts that organisms will balance their energy allocation among maintenance, storage, growth and reproduction [40,41]. While we lack direct measures of energy use and allocation, it is possible that during the recovery period, small-selected fish adopted a strategy where they allocate more and/or more efficiently energy toward growth and reproduction, potentially at the cost of allocating less energy toward maintenance, accumulating fat storage and/or activity [41]. While the small- and large-selected fish had similar condition factors (*p* > 0.05), which were lower than that of random-selected fish (*p* < 0.05), which can be used as a proxy for fat storage, we did not measure their general activity. It is possible that small-selected fish are being more passive, thus allocating less energy into activity and more into reproduction than large- and random-selected fish. Furthermore, Sbragaglia *et al*. [28] showed that small-selected zebrafish had enhanced reproductive performance, particularly in terms of fertilization rate, after six generations of recovery. This was suggested to be an adaptation to increase reproductive success before harvest-induced mortality but also to enhance the rate of recovery after size-selective harvesting [28]. Differences in fertilization rate can be caused by differences in egg and sperm quality [42] and/or male spawning behaviour [43]. However, we did not measure any of these traits, and therefore, the mechanisms behind the higher fertilization rate by small-selected fish remain unknown.

While the differences in life-history traits between large- and small-selected fish had mostly eroded, random-selected fish reached a larger size and had a higher growth rate than small- and large-selected fish. They also had higher fecundity than large-selected fish. Intrinsic fecundity selection favouring large size might have operated efficiently among random fish [24], which can be expected to harbour more variation in body size compared to size-selected lines. Indeed, random-selected fish have been shown to have more phenotypic variation (measured across numerous life-history, physiological and behavioural traits) than small- and large-selected fish [44]. Furthermore, it is tempting to speculate that higher levels of phenotypic variation among random-selected fish could be explained by higher levels of genetic variation compared to size-selected lines, but we have not been able to confirm this [22]. However, epigenetic variation can also contribute to high levels of phenotypic variation [45–49]. Alternatively, it has been shown that random-selected fish are less aggressive and more social than small- and large-selected fish [27]. Although we did not measure aggressive behaviour among the selection lines, it can be speculated that lower levels of aggressiveness shown in a previous study [27] could have enhanced the growth of random-selected fish due to allocating less resources toward aggressive behaviour.

We show that many of the phenotypic differences among the zebrafish selection lines induced by five generations of size-selective harvesting were almost completely eroded after 10 generations of recovery, even though five generations of size-selective harvesting induced such large-scale genomic differences among the selection lines [9,22]. Previous studies [12,13] hypothesized that full phenotypic recovery would take three times as many generations as size-selective harvesting. However, we show that after a recovery period twice as long as the harvesting period, most of the differences in life-history traits (e.g. spawning probability, yolk and egg size, hatching time and probability) among the selection lines had eroded. Notably, it seems that reproductive traits recovered more than growth-related traits, which is in accordance with a previous study [13]. Salinas *et al*. showed that reproductive traits recovered more completely than growth-related traits, even when food availability was limited [13]. They suggested that during the non-harvesting period, traits related to high fecundity such as large egg clutches and improved larval survival are primarily under selection [13,25]. Alternatively, fecundity may be more plastic than growth throughout a fish’s lifetime, based on external factors such as a change in food availability [13,41].

Five generations of size-selective harvesting induced not only phenotypic differences among the selection lines but also large-scale genomic differences [9,22,23]. If these genomic differences were associated with the phenotypic differences, we might have expected slower recovery than 10 generations (see also Schenk *et al*. [20]). Directional selection on body size can be expected to reduce phenotypic and genetic diversity in the population, and this loss of diversity can potentially affect the ability of the population to respond to any future stressors [44]. While our study solely focussed on the phenotypic recovery, it remains to be studied whether the genomic differences induced by size-selective harvesting eroded in a similar way to the phenotypic differences. It is likely that genomic differences remained, and the populations recovered through different genetic pathways, reaching similar phenotypes [18]. For example, Sbragaglia *et al*. [31] found changed temporal patterns of diurnal swimming and feeding in their size-selected and control (random-select ed) lines, even though the molecular clock of the size-selected lines differed significantly from control lines. This suggests that phenotypic traits have pathways to buffer and mask the effect of molecular changes. Hence, it may seem that a full recovery has occurred, but changes in the environment or new selection pressures may reveal that populations’ adaptive potential has been altered.

Studies utilizing the same zebrafish selection lines have shown that after eight generations of recovery, behavioural and cognitive differences that likely arose during the five-generation-long harvesting period were still present [28,30]. Small-selected fish were found to be less active, bold and social than large- and random-selected fish and small-selected fish were also cognitively less capable than large-selected fish [27,28,30]. While we acknowledge that these differences were not monitored in fish used in our experiment, it can be speculated that there are differences in the energy allocation among the selection lines as higher cognitive skills typically correlate with a larger brain, which is costly energy-wise to produce and maintain [50]. Hence, large-selected fish may have allocated more energy into brain tissue development and less into reproduction.

While the effect of size-selective fisheries on exploited populations in nature has been studied extensively [2,4,51], the main advantage of using an experimental study is that experiments allow us to disentangle the plastic, environmentally induced and genetic changes caused by size-selective harvesting [52]. This is because, in the laboratory, we can control all confounding environmental variables [53]. However, as in any experiment with wild-originated organisms, also in our long-term experiment, the zebrafish populations experienced domestication. Zebrafish show behavioural, genetic and physiological differences from wild populations after being reared for less than 10 generations in a laboratory environment [54,55]. Our random-selected line not only served as a control line that allows us to separate changes caused by harvesting in general (non-size selective) and by the size-selective harvesting [43] but also can be used to account for the effects of domestication [25]. It is unfortunate that we only have phenotypic measurements from two time points: after the harvesting period and after the recovery period. Thus, we cannot calculate the rate of phenotypic change at each generation, which could have provided us more details about the recovery rate and its nature. Finally, it is necessary to acknowledge that experimental results cannot be directly generalized to the real fishing scenarios in nature where multiple selection pressures operating in opposite directions may be occurring simultaneously.

Our results have important implications, but other processes need also to be studied [51]. Our fish were raised in a benign environment, with ad libitum feeding and without external stressors, such as predation, parasitism and interspecific competition. In the wild, any population recovery would be affected by factors such as immigration and changes in biotic and abiotic environmental conditions such as changing temperatures and food availability [14,51]. Depleted populations could benefit from immigration as other, less-harvested populations from, for example, marine protected areas could serve as genetic reservoirs, reducing loss of genetic diversity, as size-selective harvesting typically does not induce reproductive isolation [16,56–58]. Individuals in heavily fished populations that have been selected for small body size likely have lower survival probability in harsh environments where food is limited, and predation pressure is high [7,36]. Predicting how the additional factors affect the recovery rate is difficult owing to many complex and indirect underlying mechanisms and their interactions, though our findings can provide a baseline for further investigations [51,52]. While size-selected populations seem to be phenotypically recovered, size-selection may have reduced the population’s adaptive potential, and this can further magnify the effects of a changing or stressful environment [44]. Furthermore, while we found phenotypic recovery after 10 generations, the generation time of zebrafish is quite short, being able to reproduce as young as 60 dpf [59]. This is a very short generation time compared to many commercially exploited fish species, such as cod (*Gadus morhua*), which has an estimated generation time of 7–9 years [60].

Our experiment shows that phenotypic recovery occurred after intensive size-selective harvesting. Hence, moratoria (i.e. periods with no fishing) could potentially be an effective management measure and should be considered for incorporation into the management policies of fisheries to help create more sustainable fishing practices. While we have only investigated phenotypic recovery, future work should also focus on genomic recovery and its potential effects on adaptive potential. It can be further speculated that non-size-selective fishing allows more phenotypic and genomic variation in the population, hence higher adaptive potential, and could also be a more recommendable fishing practice than size-selective one.

## Data Availability

The data, scripts and a description of them can be found at the JYX depository (jyx.jyu.fi) at [61]. The data are published under CC-BY (Deed—Attribution 4.0 International—Creative Commons). Supplementary material is available online [62].
